# Vertical Jump and Isometric Strength in Professional Female Basketball Players: Starter vs. Non-Starter Comparison

**DOI:** 10.1371/journal.pone.0344022

**Published:** 2026-03-09

**Authors:** Raúl Nieto-Acevedo, Carlos García-Sánchez, Enrique Cañadas-Garcia, Blanca Romero-Moraleda, Moisés Marquina Nieto, Dimitrije Cabarkapa

**Affiliations:** 1 Facultad de Ciencias Biomédicas y de la Salud, Universidad Alfonso X el Sabio, Avenida de la Universidad, Madrid. España; 2 Facultad de Ciencias de la Actividad Física y del Deporte, Universidad Politécnica de Madrid, Madrid, Comunidad de Madrid, Spain; 3 Department of Physical Education, Sport and Human Movement, Autonomous University of Madrid, Madrid, Spain; 4 Applied Biomechanics and Sports Technology Research Group, Autonomous University of Madrid, Madrid, Spain; 5 Department of Health, Sport and Exercise Sciences, Jayhawk Athletic Performance Laboratory – Wu Tsai Human Performance Alliance, University of Kansas, Lawrence, Kansas, United States of America; 6 D2 Lab, Novi Sad, Serbia; İzmir Democracy University: Izmir Demokrasi Universitesi, TÜRKIYE

## Abstract

This study examined whether differences in countermovement jump (CMJ) and isometric mid-thigh pull (IMTP) force-time metrics exist between starters and non-starters in professional female basketball players. Twenty-two athletes (7 starters, 15 non-starters) competing in the first Spanish basketball league completed CMJ and IMTP testing using dual force plate system. CMJ variables included jump height, peak and mean braking and propulsive force, time-to-takeoff, and net impulse. IMTP variables included peak force and rate of force development (RFD) at 0−100 and 0−250 ms. Independent t-tests and Hedges’ g effect sizes were used to assess between-group differences. No statistically significant differences were observed between starters and non-starters for any CMJ or IMTP force-time metrics of interest (p > 0.05). Both groups displayed similar values in jump height, force production, and RFD, with effect sizes ranging from small to moderate (g = 0.04–0.49). However, starters were significantly older than non-starters (p = 0.018), while no differences were found in body mass and height (p > 0.05). Overall, the findings of the present study indicate that, at the professional level of play, CMJ and IMTP performance characteristics are not capable of distinguishing starters from non-starters in women’s basketball. Starting status may be shaped more by competitive experience, technical proficiency, and tactical awareness. Although monitoring neuromuscular performance remains valuable, player selection and role differentiation appear to depend more on skill execution and contextual game demands than on strength characteristics alone.

## Introduction

Basketball performance depends on a complex interplay between technical skills, tactical understanding, and physical attributes [1–3]. The ability to perform explosive movements such as jumps, accelerations, and rapid changes of direction is particularly relevant at the professional level, where physical and cognitive demands are high [1,4]. Researchers and practitioners often implement a diverse combination of tests that assess sport-specific skills aimed to replicate certain basketball-specific demands (e.g., dribbling speed tests) [5], as well as physical tests (e.g., countermovement jump, linear sprint speed) [6–8]. Evidence indicates that several contextual and individual variables, such as age, sex, playing position, and competitive level, influence strength and power characteristics in basketball players [9–13]. Specifically, Cabarkapa et al. [9] have found that U18 athletes exhibited greater strength and power-producing capabilities (e.g., peak power, or mean and peak force) than U16 athletes. Moreover, Petrovic et al. [10] observed that male athletes demonstrated better performance in several force-time metrics during the concentric phase of the countermovement vertical jump (CMJ), including impulse, peak velocity, and mean power, as well as vertical jump height when compared to their female counterparts. In addition, in a follow-up investigation, Cabarkapa et al. [11] reported that centers generally exhibit higher CMJ force and power outputs than guards.

However, neuromuscular assessments have been less frequently employed to compare starters and non-starters in basketball. Investigations in handball [14], volleyball [15], and rugby [16] have explored whether vertical jump characteristics can help distinguish starters from non-starters. Among these, Radovic et al. [14] found that starters attained superior performance within the eccentric phase of the CMJ when compared to non-starters, when examining a cohort of professional female handball players. Nevertheless, both Chiwaridzo et al. [16] and Cabarkapa et al. [17] reported no significant differences in CMJ performance between starters and their substitutes in male rugby players and female volleyball players, respectively. Despite the rapid growth and increasing competitiveness of the women’s game, there is still a notable lack of investigations examining neuromuscular performance characteristics between starters and non-starters in women’s basketball [18].

The CMJ is widely used to evaluate lower-body neuromuscular performance in basketball [19]. Jump performance is most commonly quantified using force platforms, which provide reliable and time-efficient data while avoiding invasive measurement techniques [20]. Although CMJ testing has been established across a variety of sports [21], it has been suggested that an athlete’s rate of force development (RFD), the ability to rapidly generate high force outputs, may be a strong indicator of sport performance [22–24]. Accordingly, incorporating assessments that specifically capture RFD may provide additional insights into the physiological profile of basketball athletes. The isometric mid-thigh pull (IMTP) is a reliable and accurate test for measuring peak force production and RFD across multiple time intervals [24]. Increasingly, the IMTP has been adopted in both laboratory-based and applied sport science settings [25–27], including basketball [24,28]. Together, CMJ and IMTP assessments are commonly used in basketball to monitor changes in neuromuscular performance across a season [29], evaluate fatigue-induced performance decrements (e.g., after games or practices) [30,31], and differentiate players based on strength characteristics [32].

Based on the aforementioned research studies, there is a lack of scientiﬁc literature that uses in-depth CMJ and IMTP force plate assessment to differentiate between starters and non-starters in female basketball players, especially at the professional level of competition. Therefore, the purpose of the present study was to identify whether force-time metrics obtained via CMJ and ITMP may distinguish starters from non-starters at the professional level of basketball play. Based on the currently available literature, it was hypothesized that none of the dependent variables would differ significantly between groups.

## Materials and methods

### Participants

Written informed consent was obtained from all participants prior to data collection. The study was approved by the University Alfonso X el Sabio (2982024) and conducted in accordance with the Declaration of Helsinki. Twenty-two professional female basketball players (x̄ ± SD; height = 181.8 ± 0.1 kg, body mass = 73.7 ± 8.8 kg, age = 25.2 ± 4.2 years) volunteered to participate in the present investigation. The data was collected between 25/10/2024 and 10/9/2025. Players who were included in the starting lineup for more than 75% of the total games played across the full season [20,38] were classified as starters (n = 7), while the remaining players were classified as non-starters (n = 15). The cohort of athletes encompassed two basketball teams competing at a similar level of play in Spain (e.g., Liga Endesa) during a single competitive season. All athletes were: i) free of musculoskeletal injuries, ii) were granted permission to participate in team activities by their respective sports medicine staff, iii) participated in structured strength and conditioning training sessions more than two times per week at least six months prior to the data collection.

### Procedures

Athletes were instructed to abstain from training for 48 hours prior to testing and to maintain consistent fluid and dietary intake on the testing day, similar to their normal skill-training routine. Testing was conducted during the preseason period, beginning with the CMJ protocols followed by the IMTP test. Before testing, athletes completed a standardized warm-up protocol consisting of activation and mobilization exercises (e.g., bodyweight lunges, squats), along with low-level plyometric drills that replicated their standardized pre-training warm-up routine. Additionally, progressive standardized warm-up protocols were applied before each test to minimize potential confounding variables and enhance measurement reliability.

For the CMJ testing modality, athletes were instructed to jump “as high and as fast as possible”. CMJs were performed with the hands on the hips (i.e., no arm swing), and the countermovement depth was self-selected by the subjects to maximize CMJ height and ecological validity. Each athlete performed three jump trials. To minimize fatigue-induced performance changes, each jump was separated by a 10–15s rest interval. The mean performance of the three trials for CMJ was used for further analysis. CMJ data were collected using a portable dual force platform sampling at 1000 Hz using Hawkin Dynamics (Westbrook, ME, USA). The force platform was interfaced with a tablet to allow for direct measurement of force-time characteristics via a validated software interface (Hawkin Capture Version 8.6.1) [33]. Prior to the onset of the countermovement, subjects remained stationary on the force platform for three seconds to enable an accurate measurement of body mass. While different researchers choose different means of quantifying and labeling CMJ phases, in this study, in line with manufacturer guidelines, the CMJ was divided into an unweighting phase, braking phase, propulsive phase, flight phase, and landing phase [34,35].

Isometric strength was assessed using the IMTP on a portable force platform sampling at 1000 Hz (Hawkin Dynamics, Westbrook, ME, USA). Subjects adopted self-selected knee and hip angles (knee = 130–150°, hip = 140–160°), consistent with previous research [36]. Following the same standardized warm-up used for CMJ testing, athletes completed two submaximal IMTP trials at 50% and 75% of perceived maximal effort [37]. Maximal IMTP trials were performed with lifting straps to prevent grip loss [37]. The bar was attached to a chain anchored to a wooden platform, allowing height adjustments to accommodate athletes of different statures. Each subject performed two maximal IMTP trials, instructed to pull against the bar as forcefully and rapidly as possible while simultaneously driving the feet into the force plate, a cue shown to optimize testing outcomes [38]. Each maximal effort lasted five seconds, with two minutes of rest between trials. The mean of the two trials was used for subsequent analysis. Strong verbal encouragement was provided during all maximal attempts.

### Variables

Force-time metrics selected were then automatically calculated by the software using a forward dynamics approach: jump height (i.e., calculated from takeoff velocity), peak and mean propulsive force, peak and mean braking force, time to take-off, and propulsive and braking phase duration, braking and propulsive net impulse (Table 1) [39]. Peak force during the IMTP was defined as the highest absolute force recorded during the trial. RFD was calculated at two intervals: 0–100 ms and 0–250 ms, representing early and late phases of rapid force production, respectively. RFD values were derived as the average slope of the vertical ground reaction force from contraction onset to 100 ms and 250 ms post-onset [40], providing a more detailed assessment of the athletes’ explosive strength capabilities. Additional information pertaining to data analysis procedures can be found at https://www.hawkindynamics.com/hawkin-metric-database.

**Table 1:** Selected force-time countermovement vertical jump variable calculations.

Variables	Calculation
Jump height (cm)	The change in center of mass position between the instant of take-off and peak positive vertical displacement of the center of mass.
Jump momentum (kg·m·s^-1^)	The vertical momentum of the system center of mass at the instant of take-off.
Peak braking force (N)	The peak instantaneous vertical ground reaction force applied to the center of mass during the braking phase.
Peak propulsive Force (N)	The peak instantaneous vertical ground reaction force applied to the center of mass during the propulsive phase.
Braking phase (s)	The time taken to complete the braking phase.
Propulsive phase (s)	The time taken to complete the propulsive phase.
Time-to-takeoff (s)	The total time taken from the onset of movement to the instant of take-off.
Net braking Impulse (N·s)	The net vertical impulse applied to the center of mass during the braking phase.
Net propulsive Impulse (N·s)	The net vertical impulse applied to the center of mass during the propulsive phase.

### Statistical analysis

Shapiro–Wilk test and Q–Q plots corroborated that the assumption of normality was not violated. Independent t-tests were used to examine statistically signiﬁcant differences in each CMJ force-time metric between starters (n = 7) and non-starters (n = 15). Due to the within-group sample size (n < 20), Hedge’s g was used to calculate the magnitude of between-group differences (g = 0.2-small effect, g = 0.5-moderate effect, g = 0.8-large effect) [41]. Statistical signiﬁcance was set a priori to p < 0.05. All statistical analyses were completed with JASP (JASP Team, version 0.17.3).

## Results

Descriptive statistics, means and standard deviations (x̄ ± SD), for each dependent variable are presented in Table 2 (anthropometric and comparison statistics) and Table 3 (CMJ and IMTP force-time metrics and comparison statistics). Due to no statistically significant differences being found in body mass between the groups (p = 0.777; g = 0.132), the dependent variables were reported in absolute terms. Significant differences were found between groups in age (p = 0.018; g = 1.175), No statistically significant differences were found in any force-time metrics of interest between starters and non-starters (p > 0.05), including both CMJ and IMPT testing modalities.

**Table 2:** Anthropometric characteristics (x̄ ± SD) and comparison statistics (starters vs. non-starters).

Variable (unit)	All players	Starters	Non-Starters	p-value	ES
Height (cm)	181.8 ± 4.2	180.4 ± 4.0	182.5 ± 7.3	0.500	0.132
Body mass (kg)	73.7 ± 8.9	72.9 ± 8.5	74.1 ± 9.3	0.777	0.132
Age (years)	25.2 ± 6.4	28.1 ± 4.3	23.8 ± 3.3	0.018*	1.175

Note: *signiﬁcantly different when compared to starters (p < 0.05); ES – effect size.

**Table 3:** Countermovement vertical jump and isometric mid-thing pull metrics (x̄ ± SD) comparison between starters and non-starters.

Variable (unit)	All players	Non-starters	Starters	p-value	ES
Jump height (cm)	30.0 ± 4.0	30.7 ± 4.2	28.7 ± 3.5	0.295	0.492
Jump momentum (kg·m·s^-1^)	181.8 ± 23.6	184.3 ± 22.4	176.5 ± 26.9	0.481	0.328
Peak braking force (N)	1,883.6 ± 281.6	1,917.9 ± 295.6	1,810.1 ± 254.5	0.417	0.379
Peak propulsive force (N)	1,893 ± 295.1	1,928.4 ± 309.5	1,817.1 ± 267.4	0.423	0.374
Braking phase (s)	0.14 ± 0.03	0.14 ± 0.03	0.14 ± 0.02	0.876	0.072
Propulsive phase (s)	0.25 ± 0.04	0.25 ± 0.04	0.25 ± 0.04	0.697	0.181
Time-to-takeoff (s)	0.72 ± 0.08	0.71 ± 0.07	0.73 ± 0.09	0.941	0.035
Braking net impulse (N·s^-1^)	95.2 ± 16.0	95.6 ± 13.2	94.3 ± 22.1	0.865	0.079
Propulsive net impulse (N·s^-1^)	183.3 ± 23.7	185.9 ± 22.5	177.9 ± 27.0	0.474	0.334
Peak force (N)	2,372.1 ± 296.1	2,349.2 ± 311.7	2,421.3 ± 275.7	0.607	0.239
Net peak force (N·s^-1^)	1,523.8 ± 254.3	1,502.1 ± 261.9	1,570.1 ± 250.2	0.572	0.263
RFD 0–100ms (N·s^-1^)	5,494.6 ± 2,900	5,400.7 ± 3,266	5,695.7 ± 2,153.7	0.831	0.099
RFD 0–250ms (N·s^-1^)	4,225.8 ± 750.8	4,199.7 ± 709.9	4,281.7 ± 889.9	0.818	0.107

*Note: RFD – rate of force developement.*

## Discussion

The aim of this study was to examine potential differences in CMJ and IMTP force-time metrics between starters and non-starters in professional basketball. The findings showed that starters and non-starters demonstrated similar neuromuscular characteristics across both CMJ and IMTP assessments. Specifically, no statistically significant differences were observed in any of the key CMJ or IMTP variables, including peak and mean propulsive force, RFD 0−100 ms, and RFD 0−250 ms. Jump height and jump strategy metrics (e.g., time to take-off) were also comparable between groups, with most effect sizes ranging from small to moderate (g = 0.04–0.49). The only significant difference observed was that starters were older than non-starters, while no group differences were found in other anthropometric measures.

Previous literature has shown that differences in physical performance between starters and non-starters are often minimal, which seems to be in the agreement with the findings of the present investigation [14–16,42,43]. Evidence from collegiate and youth team sports supports the notion that playing status does not necessarily correspond to superior physical or neuromuscular qualities [44,45]. Fry et al. [44] and Gabbett et al. [45] observed comparable outcomes between starters and non-starters across several performance test (e.g., vertical jump, sprint or agility). Notably, previous studies have also reported no meaningful differences in age, height, or weight between starters and non-starters [15]. In contrast, the present study identified a significant age difference, with starters being older than non-starters. Taken together, these results suggest that starting status at the professional level is not primarily determined by anthropometric or physical performance characteristics. Instead, the observed age difference may reflect greater competitive experience and superior technical-tactical knowledge of the game, which are likely critical factors in distinguishing starters from non-starters [15].

The absence of significant differences in lower-body strength and power metrics between starters and non-starters in the present study may also reflect the relative homogeneity of professional female players, who undergo similar long-term training and must meet high neuromuscular standards to compete at this level [46,47]. Still, Cormie et al. [48] noted that beyond a certain threshold, further increases in strength may not provide additional performance benefits if other factors, such as technical execution or tactical understanding, are limiting. Indeed, playing time and starting status in basketball are shaped by multiple determinants, including shooting accuracy, defensive positioning, decision-making, and tactical fit [49,50]. Hoare et al. [51] found that skill-related indicators (e.g., shooting percentage, turnovers, assists) were stronger predictors of playing time than physical fitness in elite female athletes. Similarly, Conte et al. [48] reported that game-related statistics (e.g., efficiency ratings, points, assists) were more predictive of starter versus bench status than neuromuscular test results. Overall, these findings suggest that while neuromuscular qualities are essential for overall preparedness, role differentiation at the professional level is often determined by skill execution and contextual performance demands rather than isolated physical metrics. This directly aligns with the Hoffman et al. [52], who found that a large portion of athlete selection is influenced by coaches’ subjective evaluations, underscoring the importance of talent identification and on-court skills. Additionally, Gómez et al. [53] reported that tactical roles and situational strategies (e.g., opponent style, positional needs) often dictate rotation patterns more than physical characteristics.

When examining the magnitudes of force–time metrics, values observed in this investigation were comparable to those reported in previous studies on female basketball players. Philipp et al. [8], for instance, found moderate to large effect sizes favoring high-minutes players over low-minutes players in jump height (34.1 vs. 28.1 cm). Similar values have been reported for jump height (31.1 vs. 30.4 cm) and time to take-off (0.71 vs. 0.72 s), suggesting performance similarities among female athletes across cohorts. However, Philipp et al. [8] reported greater braking and propulsive net impulse values, which may be partly explained by higher body mass in their sample (86.6 vs. 73.7 kg), since greater mass increases the mechanical demands for both deceleration and propulsion (i.e., impulse = force × time). Furthermore, CMJ force-time metrics (e.g., peak and mean propulsive force, jump height) in this investigation exceeded those reported by Cabarkapa et al. [9] who focused on studying U16 and U18 top-tier female basketball players. Marked differences have also been noted between professional male and female players in concentric peak force (2.573,6 vs. 1.893,0 N), highlighting potential sex-related variations in neuromuscular performance. In addition, although Comfort et al. [54] reported no meaningful sex differences in relative peak force during the IMTP across collegiate and semi-professional team-sport athletes, Townsend et al. [24] observed significantly higher IMTP peak force in NCAA Division-I male basketball players compared with females (2,534.1 vs. 1,248 N). Interestingly, female basketball players in the current sample achieved an average IMTP peak force of 2,372.1 N, a value comparable to a NCAA Division-I male players in Townsend et al. [24]. While variations in reported force–time characteristics across studies are likely influenced by differences in competitive level, as consistently highlighted in previous literature [55], the findings of the present study suggest that the ability to produce high levels of maximal force may represent a requisite physical quality for performance at the professional level, irrespective of sex. Furthermore, these findings underscore the importance of systematic, well-designed resistance training programs in female basketball players, particularly given that this population remains relatively underserved and understudied within the strength and conditioning literature.

Previous research suggests that in women’s basketball, technical and tactical proficiency may be more decisive for role differentiation than absolute neuromuscular performance. Gonzalez et al. [56] emphasized the multifactorial nature of the sport in explaining differences between starters and non-starters across NCAA Division-I season. Similarly, Erculj et al. [55] highlighted that although physical attributes such as speed, agility, and explosive strength are important, success among elite female players is often more strongly associated with tactical decision-making. These findings align with literature on game-related statistics, where metrics such as efficiency ratings, assists, and shooting accuracy better discriminate competitive outcomes than isolated physical performance measures [49]. Collectively, this evidence supports the notion that “basketball IQ”, the ability to interpret the game, anticipate actions, and make effective decisions, plays a central role in determining starting status in women’s professional basketball.

The findings enhance current knowledge of basketball performance characteristics, yet some constraints inherent to the study design should be recognized. First, position-specific differences were not considered, which may influence force-time characteristics in basketball players, and sample size could have been larger. Second, although all participants competed at the professional level, differences associated with team competitive level as reflected by league standings may exist, potentially limiting the generalizability of the findings. Third, external load and injury history were not accounted for, both of which could have impacted performance outcomes. Finally, coaching strategies and rotations, which vary considerably on team-to-team basis, were not examined and may have influenced starter versus non-starter status.

## Conclusions

In professional female basketball players, starters and non-starters demonstrated similar CMJ and IMTP characteristics. This indicates that playing status is influenced more by technical execution, tactical decision-making, and experience than by strength or power alone. CMJ and IMTP tests remain useful for monitoring performance and adaptation but should be interpreted within a broader sport-specific framework.

## Supporting information

S1 File Inclusivity Statement. (PDF)
